# Effects of Light Spectra on Morphology, Gaseous Exchange, and Antioxidant Capacity of Industrial Hemp

**DOI:** 10.3389/fpls.2022.937436

**Published:** 2022-06-02

**Authors:** Xia Cheng, Rong Wang, Xingzhu Liu, Lijuan Zhou, Minghua Dong, Muzammal Rehman, Shah Fahad, Lijun Liu, Gang Deng

**Affiliations:** ^1^College of Agriculture and Life Sciences, Kunming University, Kunming, China; ^2^School of Agriculture, Yunnan University, Kunming, China; ^3^College of Plant Science and Technology, Huazhong Agricultural University, Wuhan, China; ^4^Hainan Key Laboratory for Sustainable Utilization of Tropical Bioresource, College of Tropical Crops, Hainan University, Haikou, China; ^5^Department of Agronomy, The University of Haripur, Haripur, Pakistan

**Keywords:** antioxidant capacity, gaseous exchange, industrial hemp, light quality, morphophysiological traits

## Abstract

One of the most important growth factors in cannabis cultivation is light which plays a big role in its successful growth. However, understanding that how light controls the industrial hemp growth and development is poor and needs advanced research. Therefore, a pot study was conducted to investigate the effects of different colors of light, that is, white light (WL), blue light (BL), red light (RL), and 50% red with 50% blue mix light (RBL) on morphology, gaseous exchange and antioxidant capacity of industrial hemp. Compared with WL, BL significantly increase hemp growth in terms of shoot fresh biomass (15.1%), shoot dry biomass (27.0%), number of leaves per plant (13.7%), stem diameter (10.2%), root length (6.8%) and chlorophyll content (7.4%). In addition, BL promoted net photosynthesis, stomatal conductance, and transpiration, while reduces the lipid peroxidation and superoxide dismutase and peroxidase activities. However, RL and RBL significantly reduced the plant biomass, gas exchange parameters with enhanced antioxidant enzymes activities. Thus, blue light is useful for large-scale sustainable production of industrial hemp.

## Introduction

Industrial hemp (*Cannabis sativa* L.) is an ancient and versatile crop valued for its uses in food, fiber, and medicinal industry (Rehman et al., 2021a). Environmental stress lead to slower growth of hemp (Jiang et al., 2021). Therefore, it is necessary to find out most favorable conditions for optimal production of industrial hemp.

Light is one of the essential environmental factors for plant growth and development. Varying light quality affects the plant growth (Rehman et al., 2017, 2020). During photosynthesis, green plants capture light energy and transform it into chemical energy (Fukuda et al., 2008). However, shifting wavelengths can affect plant morphology, anatomy, and physiology, identified by phytochromes (Kami et al., 2010; Saleem et al., 2019). For example, The blue and red light wavelengths are identified to affect many plant physiological processes during growth and development, mainly photosynthesis. (Li et al., 2020). In a previous study, blue light increased the rate of germination, leaves number per plant, number of roots, and frequency of stomata and pigment contents in *Stevia rebaudiana* Bertoni (Simlat et al., 2016). Blue light have an effect on chlorophyll biosynthesis, plant height, and stomatal opening (Heo et al., 2002; Jao et al., 2005; Shimazaki et al., 2007). Tomato grown under BL showed high yield and quality of fruit with resistance to disease (Xu et al., 2012). Blue light can also promote the accumulation of phenylpropanoid-based compounds without affecting the plant morpho-anatomical traits, while red light alters the plant morphology and physiology without showing a positive effect on secondary metabolism (Landi et al., 2020). Red light wavelength promote stem growth and flowering (Rehman et al., 2017). According to Naznin et al. (2019) Blue and red light proportion is important improve growth, pigment, and antioxidant capacity in vegetable plants under controlled conditions but the proportion of blue light with red light is species dependent. Furthermore, previous literature suggests species-specific response toward light stress in plants (Cope and Bugbee, 2013; Rehman et al., 2020) and this response of plants to light is mediated by different photoreceptors. For example, cucumber is more responsive to spectral distribution than tomato in greenhouse (Trouwborst et al., 2010; Hernández and Kubota, 2012).

Light being an important environmental factor also influences the gas exchange characteristics of a plant. For example, guard cells respond directly to blue light (Mott, 2009). Red light showed higher photosynthetic activity in *B. nivea* (Rehman et al., 2020). Red light increases stomatal conductance, net photosynthesis, intercellular CO_2_, and transpiration rate, while blue light reduce these gas exchange parameters (Saleem et al., 2020). Reduction in gas exchange attributes under blue light revealed a reduction in photosynthesis associated with stomatal closure, thus reducing transpiration rate and intercellular CO_2_ (Hogewoning et al., 2010).

Stressful environmental conditions trigger excessive production of reactive oxygen species (ROS), such as superoxide radical (O^2−^), hydrogen peroxide H_2_O_2_, hydroxyl radicals (OH), and singlet oxygen (O_2_; Maurya et al., 2020; Turan, 2020; Rehman et al., 2021b). According to Asada and Takahashi (1987), 1 % of oxygen is diverted to produce ROS in plants. ROS can damage cellular components through oxidation of carbohydrates, lipids, proteins, and DNA which cause plant death (Shah et al., 2019; Bhuyan et al., 2020). To avoid this loss, plants regulate the production of ROS by recruiting enzymic and non-enzymic antioxidants (Kurutas, 2016; Sachdev et al., 2021). Antioxidant enzymes activity under varying light spectra is more complex for example callus grown in dark showed higher antioxidant enzymes activities (Adil et al., 2019). Exposure of higher proportion of blue light with red light caused an increase in antioxidant activity in lettuce (Son and Oh, 2013). Blue light showed significantly greater antioxidant enzymes activity in *Anoectochilus roxburghii* (Ye et al., 2017). Similar observations of higher SOD and CAT activity in tomato leaves were recorded under blue light as compared to red light (Kim et al., 2013). Conversely, the antioxidant activity in *Rubus hongnoensis* was promoted under red light treatment (Oh et al., 2021). Accumulation of MDA and proline contents in leaf indicate oxidative stress (Deng et al., 2012; Rehman et al., 2019). Proline protects the plants from stresses as well as helps to recover from stress (Hayat et al., 2012). Above literature suggests that light quality evoke diverse morpho-physiological response in plants with contrasting results. Therefore, impacts of different color light wavelengths on plant response and their underlying mechanisms remain elusive and required further investigation.

Present study was designed to investigate the effects of light colors on industrial hemp growth, gaseous exchange, and antioxidant capacity with an objective to find the best suitable light spectrum for optimized hemp production. Findings of present study would provide an improved understanding of the physiological and photosynthetic responses of hemp to light quality.

## Materials and Methods

### Plant Materials and Growth Conditions

A pot experiment was conducted under glasshouse, at Kunming University, Yunnan, P.R. China during 2020. The seeds of *C. sativa* variety Bamahuoma (BM) were sown in pots of 30 cm length and 40 cm width in triplicate. Seeds of BM were provided by the Institute of Economic Crops, Yunnan Academy of Agricultural Sciences. Every pot was filled with 15 kg of soil. The soil was collected from experimental station of Yunnan University. Physio-chemical properties of the soil were: pH 7.18, 52.65 g kg^−1^ of organic matter, 224.16 mg kg^−1^ of available nitrogen, 163.09 mg kg^−1^ of available phosphorus, and 758.30 g kg^−1^ of available potassium. After emergence, the plants were thinned to five plants of uniform size per pot and placed in natural light till their height of 10 cm, then moved under color LEDs. Treatments were different color LED lights included white light as control (WL), blue (BL), red (RL), and 50% blue with 50% red mix light (RBL). LEDs were mixed at the height of 1.5 m. Red and Blue LEDs light wavelengths were as 650 and 450 nm, respectively. Recommended rate of fertilizer NPK in 3:1:2 was applied. Removal of weeds and irrigation was done when needed. Finally, pots were placed in the cabins made of porous black sheet within the glasshouse. A humidity level of 70–90% and ambient day/night temperatures of 25°/20°C (±1°C) were maintained. Sinopharm Chemical Reagent Co., Ltd. analytical grade chemicals were used in laboratory work.

### Sampling and Data Collection

Thirty days after shifting under LED lights the young and fully expanded top leaves per treatment were collected at 09:00–10:00 AM, using liquid nitrogen, and stored at −80°C for analysis in the laboratory. Sixty days after shifting under light spectra, three plants from each treatment were harvested carefully by cutting the stems at a height of 5 cm from the soil surface. Fresh biomass per plant was calculated in grams. Total number of leaves per plant, plant height (cm), and root length (cm) were measured by meter scale. Digital vernier caliper (ST22302, SG Tools, Hangzhou, China) was used to measure stem diameter, 15 cm above the root neck. Stem and leaves were then separated and dried for 72 h in oven at 80°C to get their dry weight.

### Chlorophyll Content and Gas Exchange

Chlorophyll content of the young and fully expanded top leaf per treatment was measured using Soil Plant Analysis Development meter SPAD-502 plus (Konica Minolta, Inc., Japan) during 09:30–10:30 h. Nine leaves per treatment were used to measurement net photosynthesis (*P*_n_), stomatal conductance (G_s_), transpiration rate (*T_r_*), and intercellular CO_2_ concentration (*C*_i_), using portable photosynthesis system Li-6,400 (Li-COR, Lincoln, NE, United States) during 9:30–10:30 h.

### Antioxidant Capacity

Half (0.5) gram industrial hemp leaf samples were collected from each treatment. Superoxide dismutase (SOD) and peroxidase (POD) activities were determined following Chen and Pan (1996) and Sakharov and Aridilla (1999), respectively. Lipid peroxidation in leaves was measured by thiobarbituric acid (TBA) test, which determines the content of malondialdehyde (MDA), an end product of lipid peroxidation (Heath and Packer, 1968). Leaf proline content was measured according to the method of Bates et al. (1973).

### Statistical Analysis

The data were subjected to one-way ANOVA using Statistix 8.1 (Analytical Software, Tallahassee, United States). Least significant difference (LSD) test was applied to identify differences between means (*p* ≤ 0.05). Values shown in tables or figures are mean ± standard deviation (SD). Sigma plot software was used for the graphical presentation. Pearson’s correlation was used to quantify relationships between different variables. Pearson Correlation Coefficients and Principal Component Analysis between variables of industrial hemp were calculated using R Studio.

## Results

### Influence of Light Quality on Plant Growth

Shoot fresh biomass, Shoot dry biomass, plant height, number of leaves per plant, stem diameter, and root length were highest in plants under BL, and lowest values for the same parameters were observed under RL compared with WL (Table 1). Shoot fresh biomass and shoot dry biomasses under BL were increased by 15.1 and 27%, respectively, as compared to WL. In the same way, number of leaves per plant, stem diameter and root length were increased by 13.7, 10.2, and 6.8% under BL, respectively, as compared to WL (Table 2). Plant height was increased by 2.3% in BL treatment, which was at par with plant height under WL, while RL and RBL treatments resulted in reduction of 39.2 and 20.6% in plant height, respectively, as compared to WL. Maximum reduction of 39.7% in shoot fresh biomass, 45.5% in shoot dry biomass, 52.4% in number of leaves per plant, 36% in stem diameter, and 9% in root length were recorded under RL, compared with WL.

**Table 1:** Changes in biomass and plant height of industrial hemp grown under varying colors of light.

Light colors	Shoot fresh biomass plant^−1^ (g)	Shoot dry biomass plant^−1^ (g)	Plant height (cm)
White Light (control)	23.9 ± 1.10^b^	4.48 ± 0.09^b^	95.2 ± 3.75^a^
Blue Light	27.5 ± 1.00^a^	5.69 ± 0.14^a^	97.4 ± 4.05^a^
Red Light	14.4 ± 0.74^d^	2.44 ± 0.07^d^	57.9 ± 1.84^c^
Red Blue Light	18.4 ± 0.21^c^	3.10 ± 0.05^c^	75.6 ± 3.34^b^

Values in the table are means ± SD (*n* = 3). Different letters within a column indicate significant difference between treatments (*p* ≤ 0.05).

### Influence of Light Quality on Chlorophyll Content and Gas Exchange

**Figure 1:**
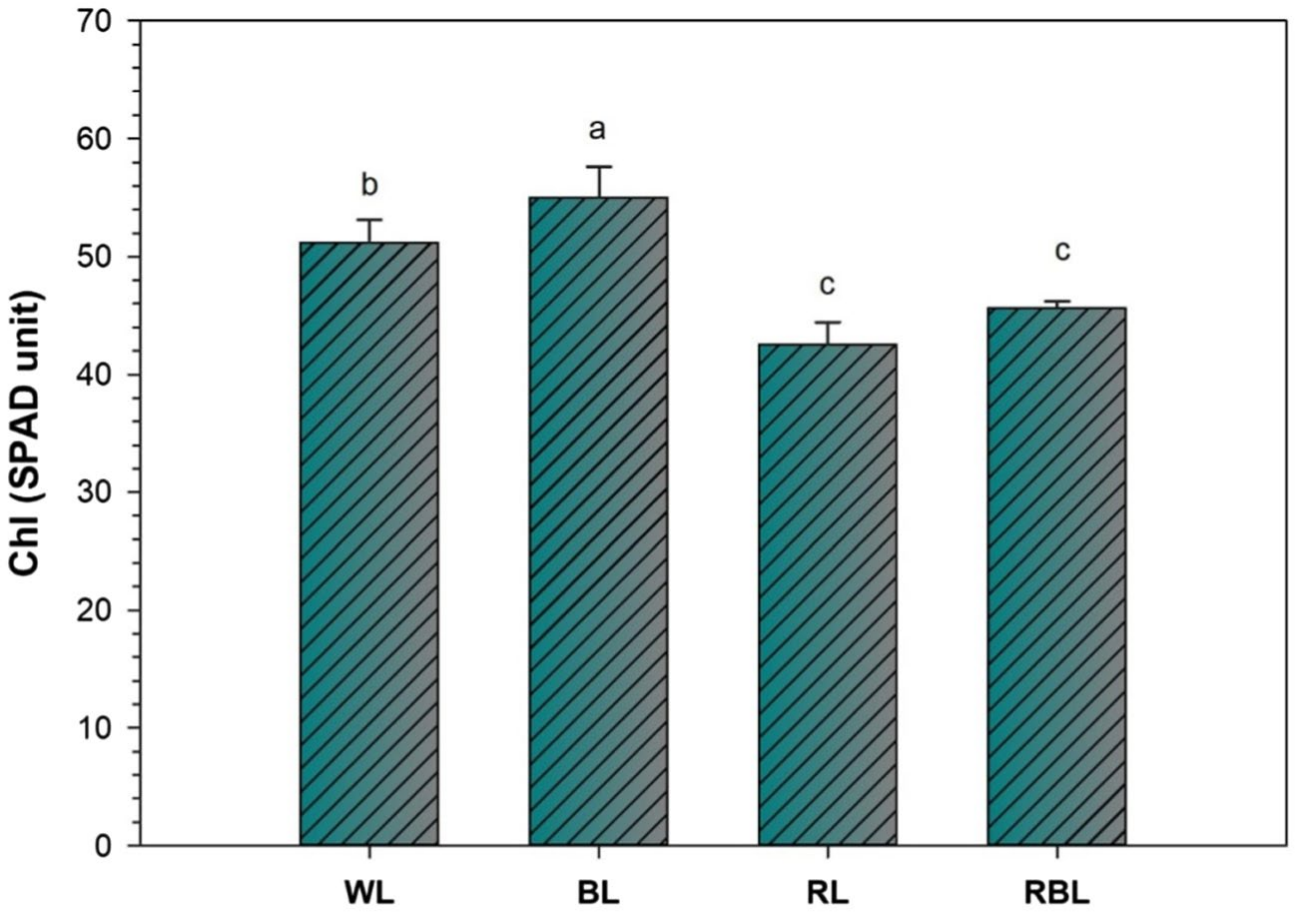
Effects of differential light quality on SPAD (Soil Plant Analysis Development) value of industrial hemp grown under different color light-emitting diodes. Bars indicated the mean ± SD (*n* = 3). Different letters on bars indicated significant difference between treatments at *p* ≤ 0.05. Different abbreviations used in the figure are as follows: WL, white light LED (control); BL, blue light LED, RL, red light LED; RBL, 50% red and 50% blue Light LED.

**Figure 2:**
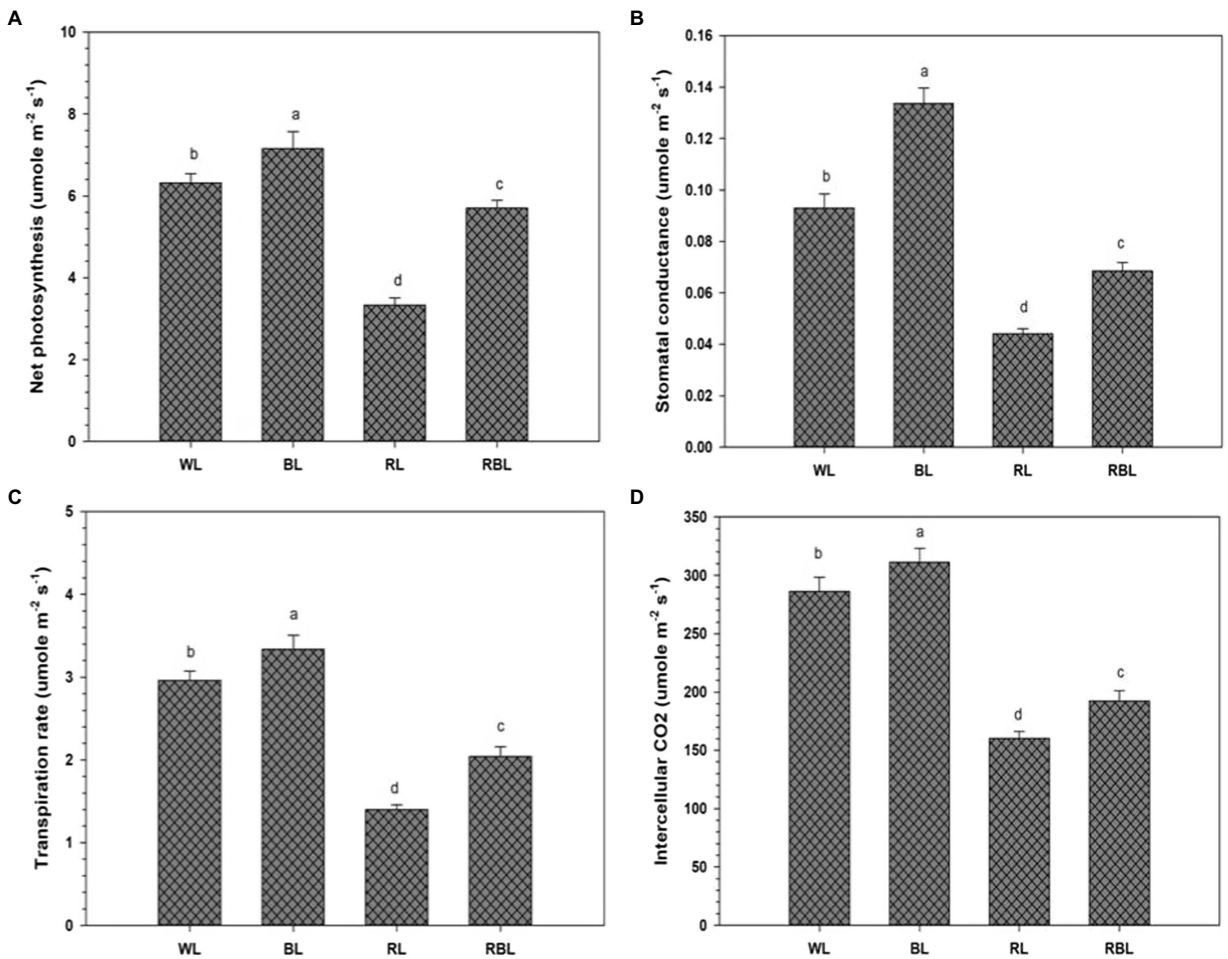
Effects of differential light quality on net photosynthesis **(A)**, stomatal conductance **(B)**, transpiration rate **(C)**, and intercellular CO_2_ concentration **(D)** in industrial hemp grown under different color light-emitting diodes. Bars indicated the mean ± SD (*n* = 3). Different letters on bars indicated significant difference between treatments at *p* ≤ 0.05. Different abbreviations used in the figure are as follows: WL, white light LED (control); BL, blue light LED, RL, red light LED; RBL, 50% red and 50% blue Light LED.

Chlorophyll change (SPAD unit) was determined in industrial hemp leaves grown under different color light-emitting diodes (Figure 1). Blue light improved the Chl contents by 7.4%; however, Chl contents were declined by 16.8 and 10.9% under RL and RBL, respectively, as compared to WL. Blue light promoted gas exchange traits in industrial hemp as represented by significant increase in net photosynthesis (*P*n), stomatal conductance (*G*s), transpiration rate (*T*r), and intercellular CO_2_ (*C*i) by 13.3, 44.1, and 12.8 and 8.8%, respectively, as compared to WL. Conversely, RL caused significant decrease in net photosynthesis (47.3%), stomatal conductance (54.2%), transpiration rate (52.7%), and intercellular CO_2_ (43.9%) compared with the WL. Furthermore, mix light RBL also showed decreased *in* gas exchange trait compared with WL (Figure 2).

### Antioxidant Capacity

**Figure 3:**
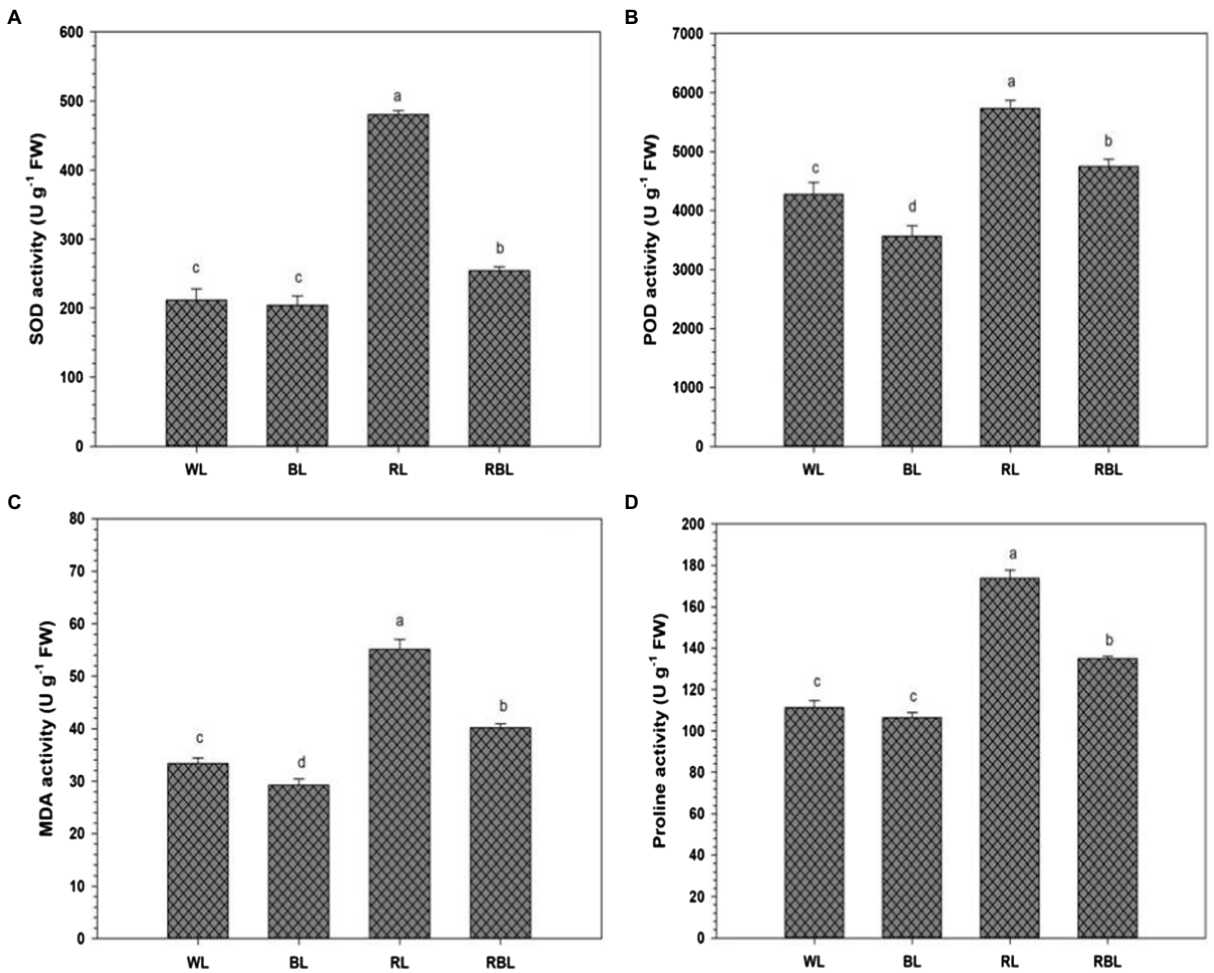
Effects of differential light quality on superoxide dismutase (SOD) activity **(A)**, peroxidase (POD) activity **(B)**, malondialdehyde (MDA) content **(C)**, and proline content **(D)** in the leaves of industrial hemp grown under different color light-emitting diodes. Bars indicated the mean ± SD (*n* = 3). Different letters on bars indicated significant difference between treatments at *p* ≤ 0.05. Different abbreviations used in the figure are as follows: WL, white light LED (control); BL, blue light LED, RL, red light LED; RBL, 50% red and 50% blue Light LED.

The activities of antioxidant enzymes superoxide dismutase and peroxidase in industrial hemp were affected by differential light quality (Figure 3). Blue light reduced POD activity by 16.6%, respectively, compared with WL. However, SOD activity under BL was statistically similar to WL. Furthermore, SOD and POD activities were increased under both RL and RBL compared with WL. Red light caused lipid peroxidation in industrial hemp, compared with WL. Malondialdehyde contents were reduced by 12.3% under BL compared with WL. Proline contents in the leaves were also reduced under BL but were statistically similar to WL. Mix light RBL resulted in increase of MDA and proline contents compared with WL.

### Correlation and Principal Component Analysis

**Figure 4:**
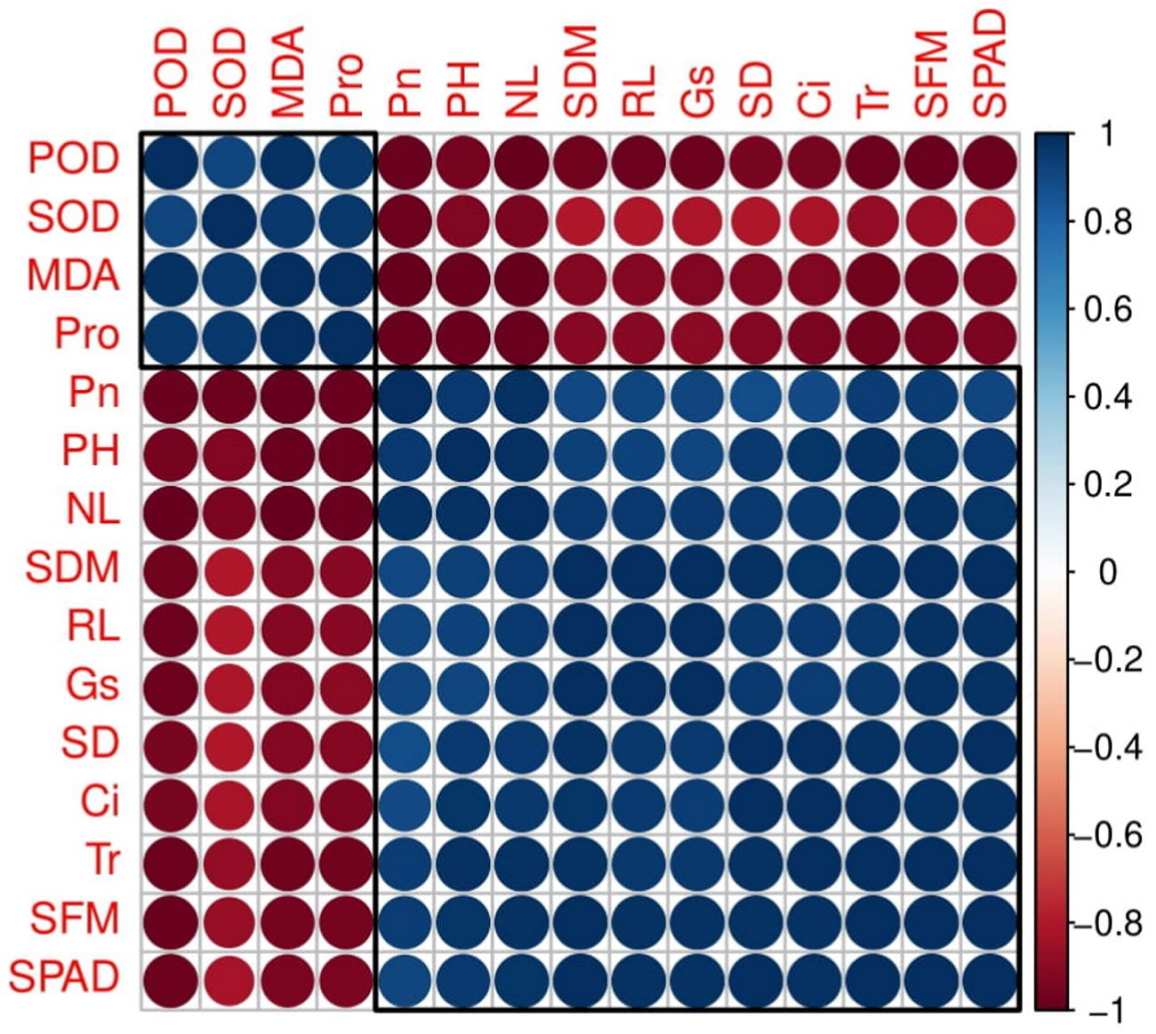
Correlation of different studied parameters in industrial hemp. Different abbreviations used in the figure are as follows: SFM, shoot fresh weight; SDM, shoot dry weight; NL, number of leaves per plant; PH, plant height; SD, stem diameter; RL, root length, SPAD, Chlorophyll; *P*n, net photosynthesis; *G*s, stomatal conductance; *T*r, transpiration rate; *C*_i_, intercellular CO_2_ concentration; SOD, superoxide dismutase; POD, peroxidase; MDA, malondialdehyde; Pro, proline.

**Figure 5:**
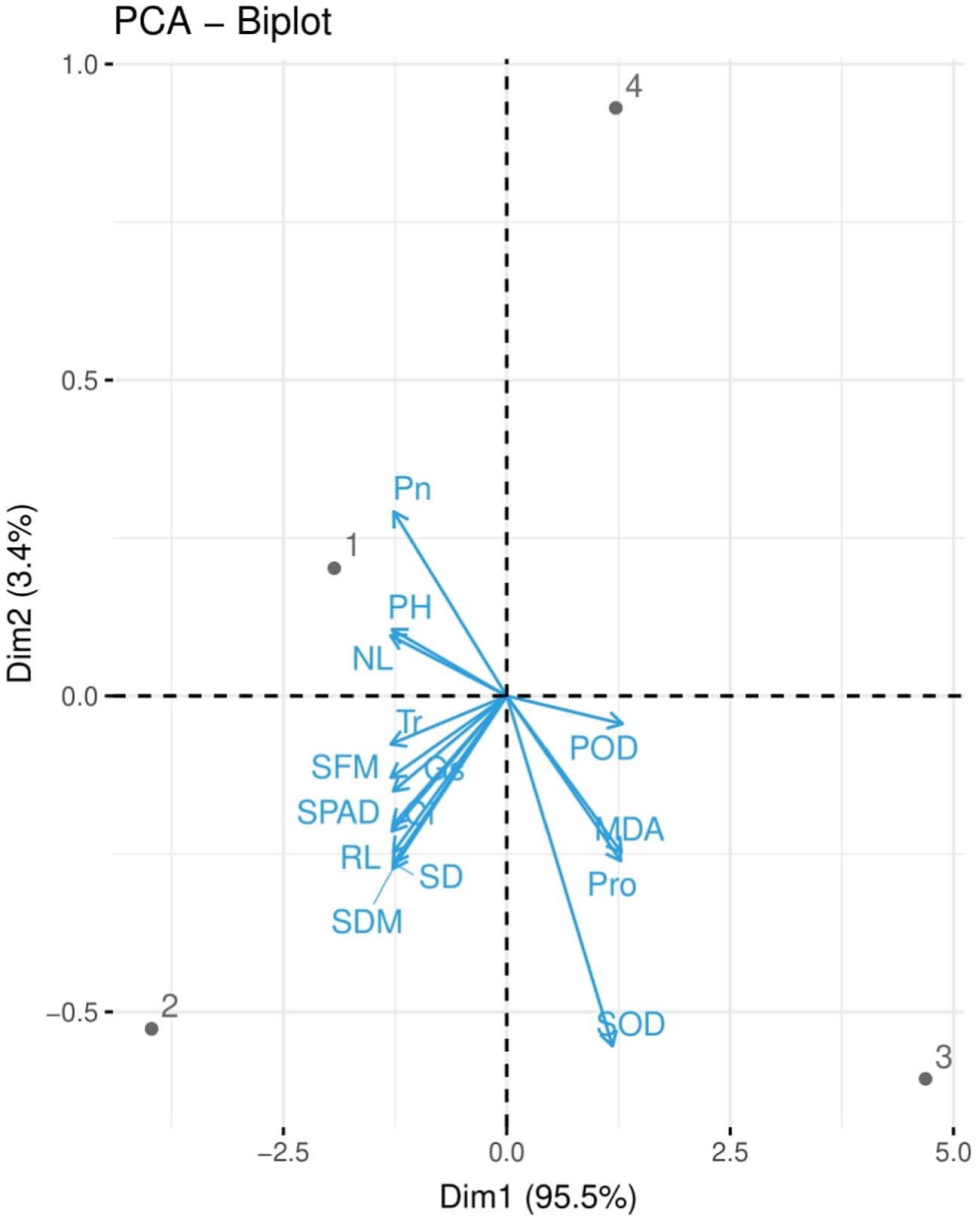
Loading plots of principal component analysis (PCA) on different studied attributes of industrial hemp grown under different color light-emitting diodes. Different abbreviations used in the figure are as follows: SFM, shoot fresh weight; SDM, shoot dry weight; NL, number of leaves per plant; PH, plant height; SD, stem diameter; RL, root length, SPAD, Chlorophyll; *P*n, net photosynthesis; *G*s, stomatal conductance; *T*r, transpiration rate; *C*_i_, intercellular CO_2_ concentration; SOD, superoxide dismutase; POD, peroxidase; MDA, malondialdehyde; Pro, proline.

Correlation between different studied parameters of plant growth, chlorophyll content, gas exchange, and antioxidant capacity of industrial hemp grown under different colors of LEDs is shown in Figure 4. Correlation showed shoot fresh biomass and shoot dry biomass, plant height, leaf number per plant, stem diameter, and root length were positively correlated with chlorophyll content, *P*n, *G*s, *T*r, and *C*i, while negatively correlated with malondialdehyde (MDA), and proline (Pro) contents and SOD and POD activities in leaves of industrial hemp. This correlation suggested a close relation between observed parameters of hemp. The loading plots of principal component analysis (PCA) to evaluate the effect of different light wavelengths on different studied attributes of *C. sativa* are presented in Figure 5.

## Discussion

Light is essential for plant growth and development (Kim et al., 2002). Plants respond to light variations for the completion of their life cycle (Ye et al., 2017). However, different colors of light influenced differently on plant growth (Fukuda et al., 2008). Seed germination, photosynthesis, biomass accumulation, stomatal opening, and closing can be optimized by adjusting light wavelengths (Taiz and Zeiger, 2002; Pinho, 2008; Vänninen et al., 2010; Saleem et al., 2019, 2020; Rehman et al., 2020).

The light-emitting diode (LED) is today’s most energy-efficient and rapidly developing lighting technology. Present study investigates the effects of different color LEDs (White, blue, red, 50% red with 50% blue mix light) on the growth, chlorophyll content, gaseous exchange, and antioxidant capacity of industrial hemp, to screen out the best suitable light spectrum higher growth of plants. Our results showed that BL significantly increase the industrial hemp growth in terms of shoot fresh and dry biomass, plant height, number of leaves per plant, stem diameter, and root length. Similar results were reported by Oana et al. (2019) that BL determined a high level of rate and fresh weight of hemp sprouts. In another study, Nanya et al. (2012) reported that stem height in tomato seedlings depend on proportion of BL. Reduction in stem height and improvement in plant extension growth was observed in cucumber grown under blue radiation which, shows a species-specific response (Hernández and Kubota, 2012). Red light and RBL reduced the growth of plants as shown by low shoot fresh biomass and shoot dry biomass, leaf number per plant, plant height, stem diameter, and root length compared with control (Tables 1, 2). Goins et al. (1997) and Yorio et al. (2001) also reported a lower dry weight accumulation in wheat, spinach, lettuce, and radish grown under red light compared with white fluorescent tubes. Thus an optimized light spectrum develop the value and quality of cannabis (Magagnini et al., 2018).

**Table 2:** Changes in number of leaves, stem diameter, and root length of industrial hemp grown under varying colors of light.

Light colors	Number of leaves plant^−1^	Stem diameter (mm)	Root length (cm)
White Light (control)	50.2 ± 1.17^b^	5.76 ± 0.17^b^	17.7 ± 1.70^b^
Blue Light	57.1 ± 1.02^a^	6.35 ± 0.29^a^	18.9 ± 3.53^a^
Red Light	23.9 ± 0.84^d^	3.69 ± 0.15^d^	16.1 ± 2.25^c^
Red Blue Light	39.4 ± 1.84^c^	4.15 ± 0.16^c^	16.8 ± 2.54^c^

Values in the table are means ± SD (*n* = 3). Different letters within a column indicate significant difference between treatments (*p* ≤ 0.05).

The blue light has been associated with leaf characteristics (Hogewoning et al., 2010). Chloroplast is an organelle where chlorophyll pigment captures light energy and converts in energy storing molecules (Kirchhoff, 2019). However, ultrastructure of chloroplast is influenced by light exposure (Chen et al., 2018). Our results revealed higher chlorophyll content in *C. sativa* leaves under BL. Similar results of higher chlorophyll content in lettuce leaves under blue light in comparison with red light were reported by (Kobayashi et al., 2013). In the current study, boost in industrial hemp biomass grown under BL might associated with higher photosynthetic rate in leaves. Blue light influenced gas exchange traits (Figure 2) and signifying an increase in photosynthesis in hemp plants. However, different species showed different photosynthetic responses under BL, and this response also vary with an increase or decrease in BL portion within a spectrum. Conversely, Matsuda et al. (2004) reported reduced photosynthetic rate in rice grown under RL, which is similar to our results. Blue light also regulates the opening and closing of stomata. Stomatal function affects the photosynthesis rate by exchange of CO_2_ and H_2_O between atmosphere and plant leaf, and the influence on stomata physiology is a well-known process regulated by BL (Lanoue et al., 2017).

Reactive oxygen species (ROS) are produced in plant cells under abiotic stress conditions. However, ROS production varies to a great extent with species of plants, their genotypes, level of stress tolerance, and the stress duration (Hasanuzzaman et al., 2020). In present study, industrial hemp grown under RL and RBL exhibit the high SOD and POD activities, while BL showed declined activity of antioxidant enzymes as compared to control (Figure 3). In contrary, a previous study on *S. rebaudiana* Bertoni showed higher POD activity when grown under BL however, RL exhibit reverse trend (Simlat et al., 2016). Uneven antioxidative response might be due to changes in protein functions in various tissues of the plants. Consequently, ROS scavenging enzymes SOD and POD are involved in the mechanisms of protection of protoplasm and cell integrity (Rehem et al., 2012). Furthermore, current research demonstrates that the plants under RL, RBL exhibit higher accumulation of MDA and proline contents, represent an oxidative damage to lipid membranes (Figure 5), while BL helps to reduce oxidative stress in industrial hemp plants. Furthermore, it was reported that hemp grown using LEDs had higher concentrations of CBD, THCV, CBG, and cannabinoids (Magagnini et al., 2018). However, the mechanisms underlying the effect of blue wavelengths on the cannabinoid pathways will require further research. Present results further confirmed that blue light benefits industrial hemp production.

## Conclusion

The results of this experiment confirm that blue light has a significant promotive effect on industrial hemp growth, biomass, gas exchange characteristics, and chlorophyll content. Moreover, blue light reduce oxidative stress on plants. However, red light and red blue mix light lessen the plant growth and photosynthesis along with higher MDA and proline contents accumulation in hemp leaves, which may damage cellular organelle membranes. Furthermore, findings of present study showed a close link between quality of light and investigated morphophysiological attributes in industrial hemp. Thus blue light can successfully be used for hemp production in industry. Keeping in view the economic significance, present technique is beneficial due to its low-cost over large-scale cultivation. However, potential of blue light spectrum for industrial hemp production should be tested at higher level.

## Data Availability Statement

The raw data supporting the conclusions of this article will be made available by the authors, without undue reservation.

## Author Contributions

LL and GD: conceptualization, supervision, and funding acquisition. XL, XC, and RW: methodology and formal analysis. LZ, MD, and MR: data analysis. MR: writing – original draft preparation. SF: writing – review and editing. All authors contributed to the article and approved the submitted version.

## Funding

This research was supported by a grant from the National Natural Science Foundation of China (31760403), Yunnan Provincial Joint Fund for Local Colleges and Universities (202001BA070001-129, 2018FH001-025, and 202001BA070001-163), the Yunnan Applied Basic Research Projects (202101AT070434), and CARS-16-E10.

## Conflict of Interest

The authors declare that the research was conducted in the absence of any commercial or financial relationships that could be construed as a potential conflict of interest.

## Publisher’s Note

All claims expressed in this article are solely those of the authors and do not necessarily represent those of their affiliated organizations, or those of the publisher, the editors and the reviewers. Any product that may be evaluated in this article, or claim that may be made by its manufacturer, is not guaranteed or endorsed by the publisher.
